# Effect of Oxidative Modification by Peroxyl Radical on the Characterization and Identification of Oxidative Aggregates and In Vitro Digestion Products of Walnut (*Juglans regia* L.) Protein Isolates

**DOI:** 10.3390/foods11244104

**Published:** 2022-12-19

**Authors:** Jinjin Zhao, Miaomiao Han, Qingzhi Wu, Xiaoying Mao, Jian Zhang, Zhenkang Lu

**Affiliations:** School of Food Science and Technology, Shihezi University, Shihezi 832003, China

**Keywords:** oxidative modification mechanism, walnut protein isolates, LC-MS/MS identification, aggregates, digestion products

## Abstract

Walnut protein is a key plant protein resource due to its high nutritional value, but walnuts are prone to oxidation during storage and processing. This article explored the oxidative modification and digestion mechanism of walnut protein isolates by peroxyl radical and obtained new findings. SDS-PAGE and spectral analysis were used to identify structural changes in the protein after oxidative modification, and LC-MS/MS was used to identify the digestion products. The findings demonstrated that as the AAPH concentration increased, protein carbonyl content increased from 2.36 to 5.12 nmol/mg, while free sulfhydryl content, free amino content, and surface hydrophobicity decreased from 4.30 nmol/mg, 1.47 μmol/mg, and 167.92 to 1.72 nmol/mg, 1.13 μmol/mg, and 40.93 nmol/mg, respectively. Furthermore, the result of Tricine-SDS-PAGE in vitro digestion revealed that protein oxidation could cause gastric digestion resistance and a tendency for intestinal digestion promotion. Carbonyl content increased dramatically during the early stages of gastric digestion and again after 90 min of intestine digestion, and LC-MS/MS identified the last digestive products of the stomach and intestine as essential seed storage proteins. Oxidation causes walnut proteins to form aggregates, which are then re-oxidized during digestion, and proper oxidative modification may benefit intestinal digestion.

## 1. Introduction

Walnuts (*Juglans regia* L.) are becoming popular as a high-quality plant-based protein with a good nutritional and health profile [1,2]. However, because walnut kernels contain high levels of unsaturated fatty acids (62–68%) [3], they are often processed into walnut oil, and the process of processing into walnut oil produces a large number of defatted walnut meals. Since defatted walnut meal contains 50–60% protein [4], it is an important source of walnut protein. In the pretreatment process of walnut oil, with the destruction of the walnut cell structure, the polyunsaturated fatty acids in walnut are prone to lipid peroxidation under the catalysis of lipoxygenase (LOX), resulting in lipid free radicals and active lipid oxidation products [5,6], which indirectly lead to protein oxidation. Moreover, walnut protein can affect the quality of the composite product as a raw material or ingredient due to its early oxidation. Oxidation first leads to changes in the structure of walnut protein, which then affects its nutritional properties. At present, the relationship between digestion and health is a hot focus of research, and walnut is also widely used in the food processing industry; therefore, research into the structure and digestive properties of walnut protein will provide a theoretical foundation for quality monitoring during the product’s processing and storage.

Protein is the material basis of life, and the absorption and utilization of nutrients by the human body are mainly carried out in the stomach and small intestine of the digestive tract. Protein source, protein modification, digestive enzyme secretion, and activity lead to differences in protein digestion and absorption [7]. Protein oxidation can lead to breakage and cross-linking of protein molecules, producing soluble and insoluble aggregates that may have effects on protein digestion, and the oxidation of amino acid side chains may cause changes in the antioxidant capacity of the oxidation products. However, the effect of oxidation on protein digestion, on the other hand, still remains debatable. Previous studies found that oxidized rice bran protein incubated with malondialdehyde has a lower digestibility index [8], whereas moderate oxidation of SPI increases its digestibility via linoleic acid modification [9]. Digestion is a complex physiological process influenced by the digestive phase’s pH, distinct oxidation systems, and degree of oxidation [10].

In the study of protein oxidative damage, the carbonyl content is often used as a key indicator of protein oxidative damage. Proteins in food are digested into oligopeptides or amino acids before being absorbed and utilized [11], which also allows the possibility of the re-oxidation of proteins during digestion [12]. Much of the current research on protein oxidation has focused on the effects of oxidation on protein structure, functional properties, and digestibility. There are few studies on the oxidative aggregation of walnut proteins and its specific effects on digestion products.

In recent years, there has been a surge of interest in the relationship between dietary protein oxidation and LC-MS/MS. Fang et al. [13] explored the effect of oxidation on protein amino acids and used LC-MS/MS to evaluate the site and mechanism of oxidation of myosin at different ozone treatment durations. Zhang et al. [14] used HPLC-HCD-MS/MS to explore the mechanism of lipid oxidation on the IgG/IgE binding capability of ovalbumin (OVA) and discovered nine oxidation sites on the α-helix of the protein. The explanation of these studies mostly starts with amino acids. However, mass spectrometry, meanwhile, has also been increasingly used for the identification of digestive products. Wang et al. [15] studied the digestibility of various proteins in the elderly gastrointestinal tract and concluded that the digestive profile of soy proteins is more susceptible to altered digestive conditions than meat proteins. Thus, amino acid analysis techniques and mass spectrometry identification techniques are important in the characterization of protein oxidation and digestion.

2,2-azobis (2-methylpropionamidine) dihydrochloride (AAPH) is frequently used as a representative of the typical free radicals of lipid peroxidation in oxidation models [16]. The formation of aggregates and their impact on digestion were investigated in this experiment by simulating in vitro AAPH oxidation and gastrointestinal digestion. To account for the digestive processes of walnut proteins, we measured the carbonyl content of proteins in different stages of gastric and intestinal digestion; we used the Tricine-SDS-PAGE profiling to characterize changes in digestion, and the digestion results were identified by LC-MS/MS. This study provides the basis for the quality control of walnut protein products. This study provides a theoretical foundation for the quality control of walnut protein products and walnut-based composite products during storage and processing.

## 2. Materials and Methods

### 2.1. Materials

Walnuts were purchased from the farmers’ market (Shihezi, China). 2,2-azobis (2-methylpropionamidine) dihydrochloride (AAPH), 2,4-dinitrophenylhydrazine (DNPH), 5,5′-dithiobis (2-nitrobenzoic acid) (DTNB), and 8-anilino-1-naphthalenesulfonic acid (ANS) were obtained from Yuanye (Shanghai, China). Pepsin (3000 units/mg) and trypsin (250 units/mg) were purchased from Boao (Shanghai, China). The Tris-Tricine-SDS-PAGE and BCA kits were provided by Solarbio (Beijing, China). All the other chemicals were of analytical reagent grade and obtained in China.

### 2.2. Preparation of Walnut Protein Isolates

The defatted walnut flour (DWF) was made using the method described by Mao and Hua et al. [3]. In brief, walnuts were shelled, peeled, and crushed, and walnut powder was soaked with n-hexane at 1:5 (*w*/*v*) for 1 h, and the precipitation was obtained after filtration. The precipitation and releaching were repeated three times. Finally, the filtered degreased walnut powder was placed in the fume hood to evaporate the organic reagent, and the dried walnut powder was sifted through an 80-mesh screen The protein content of the Kjeldahl procedure was 61.04 ± 0.13%. The walnut protein isolate was prepared according to the method described by Zhu et al. [17]. In brief, the DWF was mixed with water in a 1:26 (*w*/*v*) ratio, the pH was adjusted to 9, the mixture was stirred at 53 °C for 1.5 h, and the precipitate was centrifuged to remove the precipitate. The supernatant was adjusted to pH 4.5, stirred for 1 h, and then centrifuged to collect the precipitate. Finally, the precipitate was dissolved in deionized water, the pH was neutralized, and the solution was lyophilized at 4 °C. The Kjeldahl procedure yielded a protein content of 90.39 ± 0.30% (in the above Kjeldahl method, the conversion coefficient of protein is 6.25).

### 2.3. Modification of Walnut Proteins with AAPH

Samples of oxidized walnut protein were prepared according to and slightly modified by the method of Wang et al. [18]. Walnut protein isolates were dispersed at a concentration of 20 mg/mL in 10 mmol/L phosphate buffer (pH 8.0) (containing 0.5 mg/mL NaN_3_). They were then combined with a 1:1 mixture of walnut protein suspension and AAPH solution. AAPH concentrations in the protein solution were 0, 0.04, 0.2, 1, 5, and 25 mmol/L at the end. The resulting walnut protein solution was shaken and incubated at 37 °C under light protection for 24 h. After stopping the reaction in an ice bath, it was dialyzed for 72 h at 4 °C to remove residual AAPH before being lyophilized and stored at −20 °C.

### 2.4. Determination of Oxidation and Aggregation Formation in Walnut Proteins

#### 2.4.1. Carbonyl Group

The content of protein-bound carbonyls was determined according to the method of Wu et al. [19]. The 2,4-dinitrophenylhydrazine colorimetric method was used to measure the absorbance at 370 nm, and the molarity was calculated as 22,000/M·cm extinction coefficient per mg of protein carbonyl derivative.

(1) $$\mathrm{Carbonyl}\;\mathrm{content}\;\left(n\;\mathrm{mol}/\mathrm{mg}\right)=A\times 45.45\times \frac{D}{P\;}$$

where D is the dilution factor, P is the concentration of the protein sample in milligrams per milliliter, and A is the absorbance at 370 nm.

#### 2.4.2. Free Sulfhydryl Group

The DNTB colorimetric method was used, with slight modifications based on Fu et al. [20]. Walnut protein was dissolved in 0.1 mol/L PBS containing l mmol/L EDTA and 1% SDS pH 8.0 to fully dissolve and adjust the protein concentration. Take 3 mL protein solution, add 3 mL PBS (containing 1 mmol/L EDTA and 1% SDS), add 0.1 mL DNTB, 25 °C water bath for 1 h. After the reaction, centrifuge at 8000 r/min for 30 min. Calculate the mercapto group content by measuring absorbance at 412 nm without DNTB as control. The results were expressed as nmole of -SH per milligram of soluble protein with a molar extinction coefficient of 13,600/M·cm.

(2) $$\mathrm{Free}\;\mathrm{sulfhydryl}\;\mathrm{content}\;\left(n\;\mathrm{mol}/\mathrm{mg}\right)=B\times 70.67\times \frac{D}{P\;}$$

where D is the dilution factor, P is the concentration of the protein sample in milligrams per milliliter, and B is the absorbance at 412 nm.

#### 2.4.3. Free Amino Group

The free amino content was determined by its derivatization with OPA, according to Chen et al. [21]. Dissolve 40 mg phthalaldehyde in 1 mL methanol, 2.5 mL 20% SDS, 25 mL 0.1 mol/L borax, 100 μL β-mercaptoethanol, mix well and keep volume constant to 50 mL. Then mix 200 μL of each protein solution with 4 mL of OPA reagent, react at 35 °C for 2 min, and measure the absorbance at 340 nm. The content of the free amino group was calculated from a standard curve made from tryptophan with linearity (R^2^ = 0.9976; *p* < 0.05).

#### 2.4.4. Secondary Structure (FTIR)

The protein’s secondary structure was determined using FTIR spectrometry according to the method of Zhu et al. [22]. Add 100 mg of potassium bromide powder to the sample, mix, and tablet. The blank control was potassium bromide, and the infrared spectrometer was used to perform full-band scanning (4000–400 cm^−1^) with a resolution of 4 cm^−1^ and a total of 32 scans.

#### 2.4.5. Surface Hydrophobicity

The ANS fluorescence probe method was slightly modified from Wu et al. [23]. The protein concentrations in the samples ranged from 0.005–0.5 mg/mL. A total of 4 mL of the diluted sample was added to 50 μL of ANS solution (0.01 mol/L pH 7.0 phosphate buffer at 8 mmol/L), and the excitation and emission wavelengths were set at 395 nm and 473 nm, respectively.

#### 2.4.6. UV Spectrum Analysis

The ultraviolet (UV) spectrum was measured using the method previously described by Hellwig et al. [24] with minor modifications. The protein sample concentration was 0.5 mg/mL, and the instrument scan range was 190 nm–500 nm.

#### 2.4.7. Fluorescence Determination of Tryptophan, NFk, and Schiff Bases

Walnut protein was dissolved in phosphate buffer (10 mmol/L, pH 7.0), magnetically stirred for 2 h, and then centrifuged at 8000 rpm for 30 min at 4 °C. The protein concentration in the sample was 0.5 mg/mL. The fluorescence of tryptophan was measured using a slightly modified method developed by Wu et al. [23]. The emission spectra were scanned at 300–400 nm, using 285 nm as the excitation wavelength. The Schiff bases and N′-formyl-L-kynurenine (NFk) were determined using a slightly modified method of Fu et al. [20]. The width of the slit was 5 nm, and the voltage was 500 mV. The excitation wavelength was set to 350 nm, and the fluorescence value at 460 nm was used to calculate the Schiff base intensity, while the fluorescence value at 440 nm was used to calculate the NFk intensity.

### 2.5. Digestion In Vitro

#### 2.5.1. Simulation of the Digestive Process

By referring to Brodkorb et al. [25], the digestion protocol was slightly modified. Berify used various concentrations of KCl, KH_2_PO_4_, NaCl, MgCl_2_ (H_2_O)_6_, (NH_4_)_2_CO_3_, and CaCl_2_(H_2_O)_2_ to simulate 1.2X stomach gastric electrolyte solution (SGF) and intestinal electrolyte solution (SIF). The pH of the SGF was adjusted to 3.0 with hydrochloric acid before use, and the pH of the SIF was adjusted to 7.0 with NaOH before being mixed with the enzyme solution (4 parts electrolyte solution plus 1 part enzymes). The electrolyte solutions of SGF and SIF were preheated to 37 °C. To avoid precipitation during storage, CaCl_2_(H_2_O)_2_ should be added immediately before the digestion experiment. Gastric digestion was performed at 37 °C for 2 h with stirring at 100 rpm, with samples taken at 0, 30, 60, 90, and 120 min, labeled G-30, G-60, G-90, and G-120. The extracted samples were boiled for 5 min to inactivate the enzyme, and digestion was continued for 2 h with a 1:1 addition of intestinal digestion solution, with samples taken at 0, 30, 60, 90, and 120 min, labeled I-30, I-60, I-90, and I-120. The supernatant was collected by centrifugation at 6000 rpm for 20 min.

#### 2.5.2. Gel Electrophoresis Analysis

Follow the method of Zhu et al. [22], with minor modifications, after adding 10 μL of the same volume of 2X SDS sample buffer with β-mercaptoethanol (reducing), the samples were boiled for 5 min. Concentrates and separates were run at 80 and 120 volts, respectively, stained with R-250 Coomassie Brilliant Blue for 30 min, and then decolorized with 7.5% glacial acetic acid and 25% methanol. Following the method of Xu et al. [26] with minor modifications, a Tricine-SDS-PAGE kit was used to determine the molecular weight composition of walnut peptides from the gastric and subsequent intestinal digestion. The samples were boiled for 10 min after the 5X loading buffer with β-mercaptoethanol (reducing) was added at a volume ratio of 1:5. The gel concentrations were as follows: 16% of separation gel, 10% of interlayer gel, and 4% of concentration gel. Cathodic electrophoresis buffer was used in the inner bath, while anodic electrophoresis buffer was used in the outer bath. The voltage was set at 30 volts at the start of the experiments and increased to 90 volts once the bands were in the interlayer gel. The gel was first fixed for 1 h with fixative, then stained for 30 min with R-250 Coomassie Brilliant Blue, and finally decolored with a 7.5% acetic acid and 25% methanol solution.

#### 2.5.3. Determination of the Protein Carbonyl Indexes in Gastrointestinal Digestion

According to 2.4.1, the carbonyl values of walnut protein samples after oxidation of AAPH with different concentrations were measured, and samples were taken at 30, 60, 90, and 120 min during gastric digestion and labeled G-30, G-60, G-90, and G-120, while the samples were taken at 30, 60, 90, and 120 min during subsequent intestinal digestion and labeled I-30, I-60, I-90, and I-120.

#### 2.5.4. Amino acid Decay Rate before and after Digestion of Oxidized Protein

An automatic amino acid analyzer was used to measure 17 different amino acids (Asp, Thr, Ser, Glu, Gly, Ala, Cys, Val, Met, Ile, Leu, Tyr, Phe, Lys, His, Arg, and Pro) using the method of Kuipers and Gruppen et al. [27]. The amino acid results are expressed in grams of amino acids per 100 g of liquid supernatant.

#### 2.5.5. Determination of Antioxidant Activity of Hydrolysates

Adopting the method of Georgiou et al. [28], the ABTS free radical scavenging ability, DPPH free radical scavenging ability, and hydroxyl free radical scavenging ability of the hydrolysate were determined.

### 2.6. Identification of Oxidized Protein Digestive Products

#### 2.6.1. LC-MS/MS

##### In-Gel Digestion

Referring to the method of Fang et al. [13], with minor modifications, the gel was carefully placed into a 1.5 mL EP tube and washed twice with ultrapure water for 5 min each time before being decolorized with 400 μL (25 mmol/L NH_4_HCO_3_, 50% acetonitrile), rinsed until transparent, and freeze-dried. Lyophilized samples were hydrolyzed in 10 μL of a 20 ng/μL trypsin solution for 30 min at 4 °C. This is a pre-trypsin digestion step that completely absorbs the gel block, improving and fully hydrolyzing the gel during subsequent digestion. After being treated with 25 mmol/L of NH_4_HCO_3_ buffer, the gel was hydrolyzed at 37 °C for 16 h (without trypsin). After that, the hydrolyzed solution was extracted and transferred to a new EP tube. The original tube contained 0.1% trifluoroacetic acid (20 mL). The solution was siphoned after 15 min of incubation, added to the previous solution for 15 min of ultrasound, and then freeze-dried.

#### 2.6.2. Easy-n LC 1200 Combined with a Q-Exactive Mass Spectrometer

Reversed-phase nano-liquid chromatography–tandem mass spectrometry (LC-MS/MS) was used to analyze each sample. The peptide amino acid sequences were obtained from the Uniprot database at https://www.uniport.org/ (accessed on 11 December 2022).

### 2.7. Statistical Analysis

The data were analyzed using SPSS 26.0 statistical software, and the graphs were created using Origin 2022. To assess significant differences (*p* < 0.05), Duncan’s multiple range test was used.

## 3. Results

### 3.1. Effect of Oxidation on Protein Carbonyl Groups, Free Sulfhydryl Groups, and Free Amino Groups

Protein carbonyl groups and free sulfhydryl groups are often used to characterize the extent of protein oxidation. According to Soudani et al. [29], protein side chains (especially Pro, Arg, Lys, and Thr) readily generate carbonyl (CO) groups (aldehydes and ketones) during oxidation. Under constant rate thermal decomposition conditions, AAPH is a water-soluble azo compound that generates peroxyl radicals [30]. The amount of peroxyl radicals produced by AAPH is proportional to the concentration of AAPH [31]. As shown in Table 1, the carbonyl content increased significantly with increasing AAPH concentration (*p* < 0.05), and a similar trend was reported by Mao et al. [5]. In this study, the initial carbonyl content of the walnut sample was 2.36 nmol/mg, and the carbonyl content increased 0.35-fold and 1.17-fold at AAPH concentrations of 1 mmol/L and 25 mmol/L, respectively, compared to the control.

The free sulfhydryl content of walnut blank treated samples was 4.30 nmol/mmg, and it was already reduced by 48.37% at an AAPH concentration of 1 mmol/L. This may be because oxidation causes the unfolding of the protein structure, turning it into intramolecular and intermolecular disulfide bonds [32]. This suggests that a significant loss of free sulfhydryl groups had already occurred in walnut samples when the concentration of AAPH was 1 mmol/L and that this loss was linked to the development of protein aggregates. He et al. [33] studied the structural effect of heat treatment on quinoa and found that heat treatment increased the content of disulfide bonds and decreased the digestibility in vitro.

Another important marker of protein oxidation is amino acid composition [34]. The free amino acid content increased from 1.28 μmol/mg to 1.47 μmol/mg with increasing oxidation concentration but notably dropped at 1 mmol/L, with a fall of 23.1% from 0.2 mmol/L. The function of amino acids containing NH or NH_2_ in the side chain in carbonyl group synthesis was supported by the decline in free amino acid content, which is likewise consistent with the rise in carbonyl content [35]. The results suggested that the reduction in the amount of free amino acids in walnut samples might be caused by their interaction with an oxidizing agent to produce more adducts.

### 3.2. Changes in Protein Structure after Oxidative Modifications

**Figure 1:**
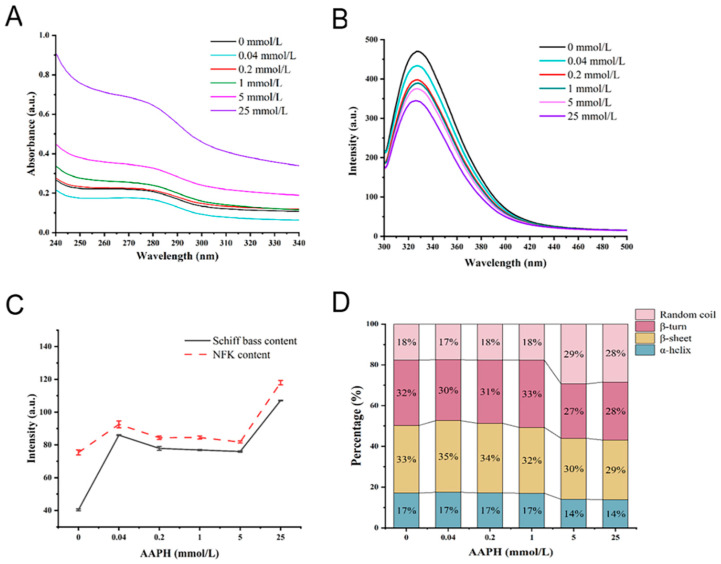
(**A**) UV absorption; (**B**) endogenous fluorescence; (**C**) Schiff base and NFk content; (**D**) secondary structure. Values of 0, 0.04, 0.2, 1, 5, and 25 represent various AAPH concentrations (unit: mmol/L), and different letters (a–d) indicate significant differences at *p* < 0.05.

Protein tertiary structure alterations are frequently studied using spectroscopy. The UV spectrum of the protein is mainly derived from the absorption of the side chains of tryptophan and tyrosine residues at the UV wavelength of 280 nm [36]. Figure 1A shows that when the AAPH concentration was 1 mmol/L, the UV absorption intensity of protein at 280 nm increased significantly, indicating that the protein structure had been disrupted.

Tryptophan is environmentally sensitive and highly susceptible to oxidation, and the endogenous fluorescence intensity typically demonstrates changes in tryptophan residues [37]. As shown in Figure 1B, the control sample had the highest fluorescence intensity of 431.55, and as the concentration of AAPH increased, the endogenous fluorescence intensity decreased to 344.88. This could be due to the low single-electron oxidation potential of tryptophan residues in the protein, which are susceptible to free radical capture and degradation to kynurenine, resulting in a decrease in the walnut protein’s endogenous fluorescence intensity [38]. In addition, tryptophan’s maximum absorption wavelength shifted blue, and the blue shift illustrates that as the concentration of AAPH increases, the non-polarity of the tryptophan microenvironment also increases, demonstrating that oxidative aggregation of the walnut protein leads to the return of unoxidized tryptophan in the protein molecule to the non-polar environment.

N′-formyl-L-kynurenine (NFk) is a tryptophan metabolism intermediate product [39]. The NFk content appeared at a higher level when the AAPH concentration was 0.04 mmol/L, as shown in Figure 1C, which may be the result of mild oxidative modification. However, the trends in NFk content and tryptophan intensity were not fully complementary, explaining that NFk is not the only tryptophan degradation product. This result is consistent with Li et al. [40] Schiff bases are organic compounds that have imine or methylimine character groups (-RC=N-) and are formed by the condensation of amines with reactive carbonyl groups [41]. In Figure 1C, Schiff bases show a similar trend to NFk, which may be due to the similar detection wavelengths of NFk and Schiff (440 nm for Schiff bases and 460 nm for NFk), and it is consistent with the findings of Xi and Chen et al. [42]. Furthermore, protein carbonyl group formation can be covalently attached to the adjacent free amino group in the lysine residue, condensing the Schiff base and causing protein covalent cross-linking, confirming that protein carbonyl group formation is a manifestation of oxidative damage and aggregation.

The effect of oxidation on the secondary structure of walnut proteins was assessed using the band of amide I (1600–1700 cm^−1^) in the protein’s FTIR spectrum. It has been demonstrated that the α-helix content in the secondary structure is closely related to protein in vitro digestibility, and that it is positively correlated with in vitro digestibility [43]. Figure 1D shows that when the concentration of AAPH was 5 mmol/L, the proteins of the α-helix were visibly reduced, while the random coil was increased in the secondary structure. This demonstrates that as oxidation levels increase, the structure of proteins changes from orderly to disorderly, which may also have some effect on protein digestion.

### 3.3. Effects of Oxidation on Protein Aggregation

**Figure 2:**
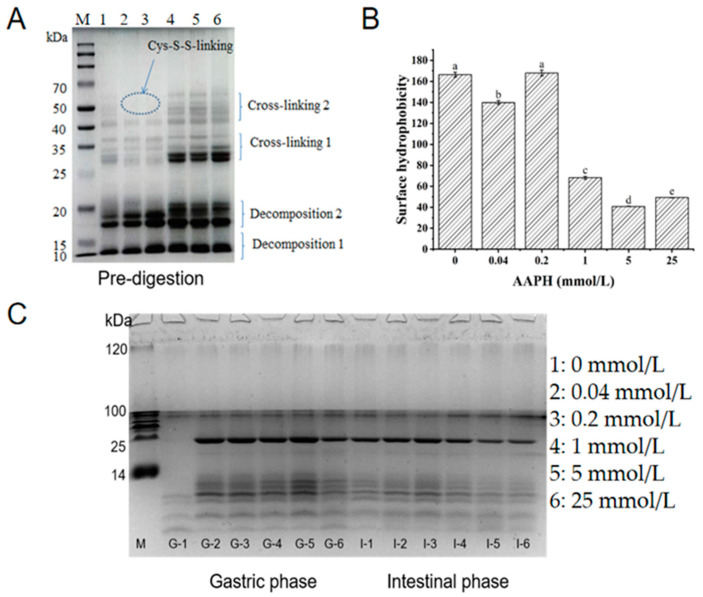
(**A**) Electrophoresis profiles of walnut isolated proteins after 24 h of incubation with different concentrations of AAPH, the circle shows the cross-linking of two sulfhydryl groups of cysteine due to oxidation; (**B**) surface hydrophobicity; and (**C**) small-molecule electrophoresis profiles of walnut isolated proteins after AAPH oxidation in gastric and subsequent intestinal digestion for 2 h, respectively, G: gastric phase, I: intestinal phase, and different letters (a–e) indicate significant differences at *p* < 0.05.

SDS-PAGE has the advantages of being fast, having a high resolution, and having high sensitivity, making it especially suitable for the determination of the molecular weight of proteins [44]. Figure 2A shows the profile of walnut protein incubated with different concentrations of AAPH, where the intensity bands of 10–15 kDa (Decomposition 1) and 17–20 kDa (Decomposition 2) deepened with increasing concentrations of AAPH (1 mmol/L), with apparent cross-linking of bands of 55–70 kDa (Cross-linking 2) and 30–35 kDa (Cross-linking 1) occurring when the concentration of AAPH was 1 mmol/L. Feng et al. [45] used reactive oxygen species-modified whey protein concentrate to conclude that mild oxidation can produce soluble aggregates, while increasing the oxidizing agents causes insoluble cross-linking. In this experiment, at the oxidizing agents of 0.04 and 0.2 mmol/L, the bands of 55–70 kDa were blurred more than in the control group, which may be the reason for having produced the soluble aggregates. On the other hand, when the oxidizing agent increased, the 55–70 kDa subunits were enhanced and the insoluble aggregates formed. The aforementioned phenomenon is consistent with the change in the free sulfhydryl group, as shown in Table 1. When AAPH was 0.5 mmol/L, free sulfhydryl decreased by 14.88%, and when AAPH was 1 mmol/L, it decreased by 48.37%. This implies that the decomposition of oxidized disulfide bonds is greater than the synthesis and decreases as the oxidant concentration increases. The formation of soluble aggregates may be caused by the Cys-S-S-linking formed by the reversible reaction of free sulfhydryl groups and cysteine.

Protein aggregates have previously been reported to form as a result of surface hydrophobic interactions [45]. As demonstrated in Figure 2B, the surface hydrophobicity falls with an AAPH concentration of 0.04 mmol/L and experiences a dramatic reduction at an AAPH concentration of 1 mmol/L. Peroxy radicals can cause the conversion of hydrophobic side-chain amino acid residues into hydrophilic groups, which can then be incorporated into the protein, resulting in the oxidation of amino acid residues in the protein and the unfolding and exposure of hydrophobic groups [37]. In Figure 1C, NFk content and Schiff base content were significantly increased when AAPH concentrations were below 1 mmol/L, indicating that tryptophan metabolism might be related to the formation of soluble aggregates. As a result of hydrophobic interactions, the exposed hydrophobic groups form aggregates, resulting in a decrease in surface hydrophobicity.

### 3.4. Protein Oxidation and Digestibility during Digestion In Vitro Simulation

The digestibility of walnut samples with varying degrees of oxidation in the process of gastric and subsequent intestinal digestion can be observed from the Tricine-SDS-PAGE profiles. Figure 2C shows that there were significant differences in the gastric phase between the control and oxidized proteins (G-1 with other channels), with the control sample (G-1) only having blurred bands with small molecules (14 kDa or less), and the band distribution of oxidized walnut proteins was extremely similar throughout the digestion time, but with an increasing intensity of bands (G-2 to G-5) with increasing oxidation degree in the gastric phase. The G-5 band is clearly deepened relative to the G-2 band as seen on the gel profile, which suggests that oxidation would have a resistant effect on the stomach due to the greater concentration of G-5 oxidation. The shallow band of the G-6 channel may be responsible for the excessive oxidation. Sante-L et al. [46] believe that oxidative aggregation of proteins reduces the recognition site of pepsin and thus reduces protein digestibility. In the intestinal phase, some interesting facts are that the intensity of the control band (I-1) is stronger than the gastric (G-1), and as the degree of oxidation increases, the bands (I-2 to I-6) become shallower. These phenomena are possibly due to the re-oxidation of the digestion phase, which can also explain the control band of I-1 with G-1, the hydrolysis of walnut in the gastric phase, and the change in sample structure with oxidation, which may result in more trypsin recognition sites and make the oxidation protein more digestible.

### 3.5. Dynamic Changes in Carbonyl Content during the Digestion of Walnut Protein

**Figure 3:**
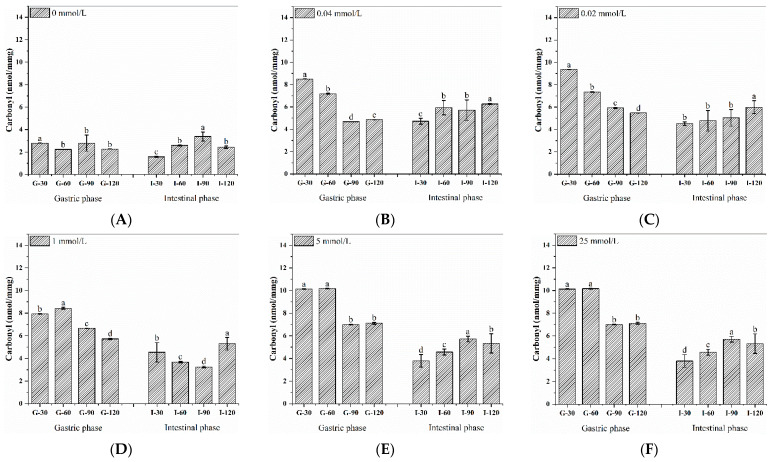
Carbonyl content of walnut isolated proteins after AAPH oxidation in the process of gastric and subsequent intestinal digestion at 30, 60, 90, and 120 min, respectively. (**A**–**F**) represent different incubation concentrations of AAPH, and different letters (a–d) indicate significant differences at *p* < 0.05.

The amount of protein carbonyls may be an indicator of oxidative protein damage in the body [47]. The presence of re-oxidation during the digestion of walnut protein and the impact of different levels of oxidation on digestion were tested using the carbonyl content during digestion. When compared to the sample’s carbonyl content without the digesting treatment (Table 1), we can see that Figure 3A–F with G-30 has a greater carbonyl content, and thus the pH is thought to be an easy trigger for the rise in oxidation-induced protein carbonyl concentration. It can also be noticed that the carbonyl content of late gastric digestion is reduced to a certain extent, which may be because the digestion concentration has changed. Additionally, after 90 min of intestinal digestion, carbonyl levels at nearly all oxidation concentrations increased. This finding may be related to the fact that the protein that is hydrolyzed into the active peptide is more likely to be oxidized. According to Van-H et al. [48], the digestion of red meat considerably raised the levels of protein carbonylation and lipid oxidation. Therefore, during digestion, proteins from both plants and meat can undergo further oxidation. Therefore, it is crucial to take into account the protein products’ active oxidation protection.

### 3.6. Antioxidant Capacity of Digestion Products

**Figure 4:**
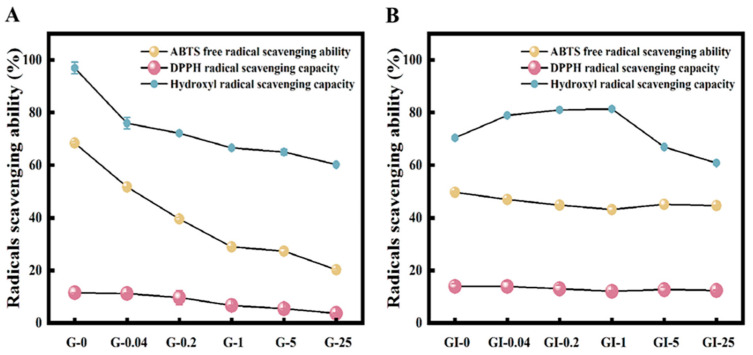
The antioxidant capacity of walnut isolated proteins after AAPH oxidation in gastric digestion (**A**) and subsequent intestinal digestion (**B**) for 2 h, respectively. G: gastric phase; GI: intestinal phase; 0, 0.04, 0.2, 1, 5, and 25 represent various AAPH concentrations (unit: mmol/L).

Figure 4 depicts the antioxidant capacity of digestion products. The gastric digestion products of walnut proteins showed reduced scavenging activity against ABTS, DPPH, and hydroxyl radicals after oxidation by peroxyl radicals. However, at mild oxidation doses, hydroxyl radicals in the intestinal phase were slightly elevated, most likely due to changes in peptide fragments. Certain amino acids and their derivatives, such as Cys, His, Try, Met, Tyr, and Pro, for example, have antioxidant properties and can effectively scavenge free radicals [49]. Furthermore, hydroxyl radical antioxidant activity was lower in ABTS after intestinal digestion than in the gastric phase, most likely because the stomach digestion comprised peptides comprising the hydrophobic amino acids alanine, valine, and leucine, which act as antioxidants by binding to oxygen.

### 3.7. Mechanism of Selective Oxidation with Amino Acids

**Figure 5:**
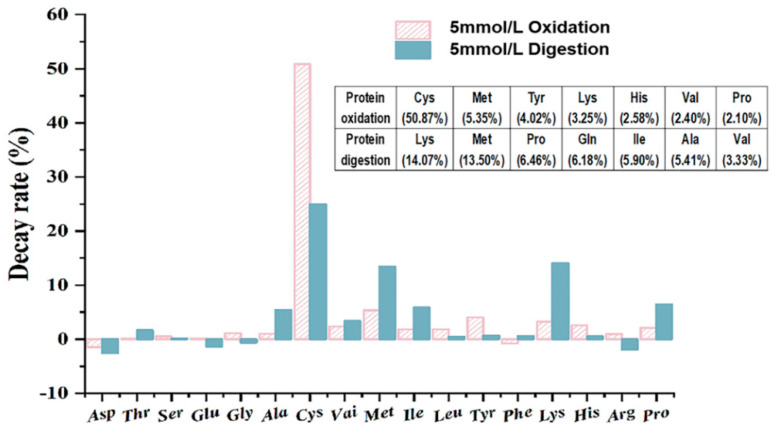
The decay rate of 17 amino acids for walnut isolated proteins after AAPH oxidation and digestion for 2 h at the concentration of AAPH is 5 mmol/L, respectively.

The quantity and composition of amino acids can affect the degree of protein absorption and utilization. Here, we have chosen a representative concentration that is typical (AAPH = 5 mmol/L). As shown in Figure 5, almost all amino acid levels were decreased to varying degrees during in vitro oxidation and digestion. The decay rates of amino acids in walnut samples before and after oxidation are ranked from high to low as: Cys, Met, Tyr, Lys, His, Val, and Pro, and the decay rates of amino acids in walnut samples before and after digestion are ordered from high to low as: Lys, Met, Pro, Gln, Ile, Ala, and Val. Fang et al. [13] showed that Cys, Met, Tyr, and Lys are vulnerable. The most frequent sites of oxidation were determined to be His, Lys, Pro, Arg, Met, and Cys by Stadtman et al. [50]. From the results of the present study, the oxidation of amino acids is selective, and cysteine is the most vulnerable amino acid and is typically the first to be oxidized, despite the fact that theoretically all amino acid side chains are vulnerable to free radical attack. The main byproduct of the oxidation of sulfur-containing amino acids such as Met is methionine sulfoxide, which accounts for the reduced Met level. Since residues such as Cys, Try, Met, Arg, and Lys are frequently the targets of free radicals, amino acids with active side chains, such as those found in sulfhydryl, thioether, amino, mitochondrial, and indole rings, are similarly vulnerable to oxidation.

### 3.8. Antidigestive Protein Identification and Peptide Sequence Alignment

**Figure 6:**
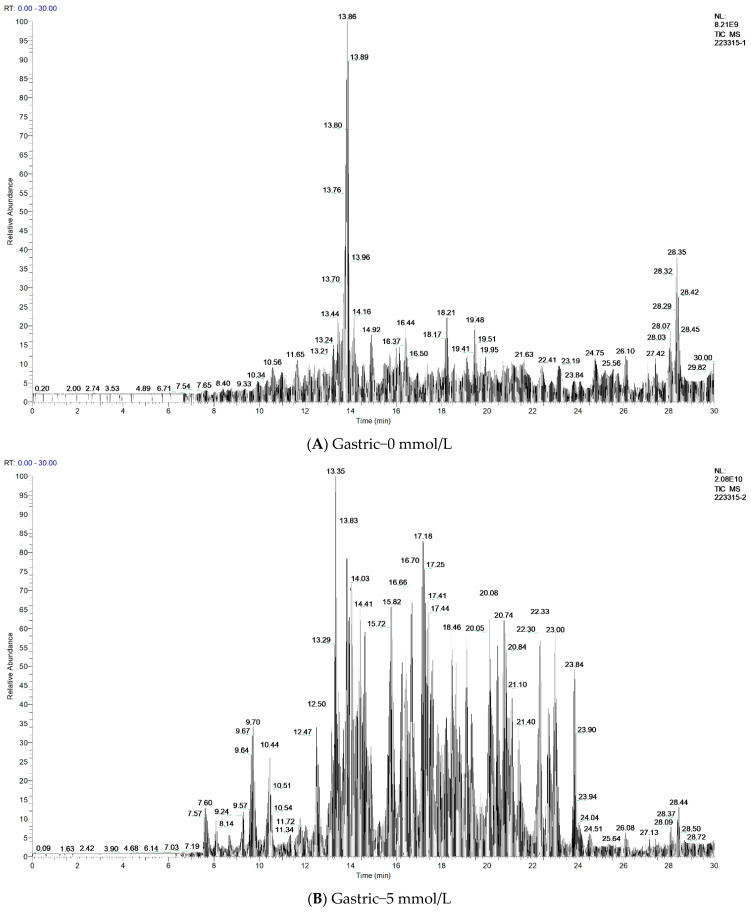

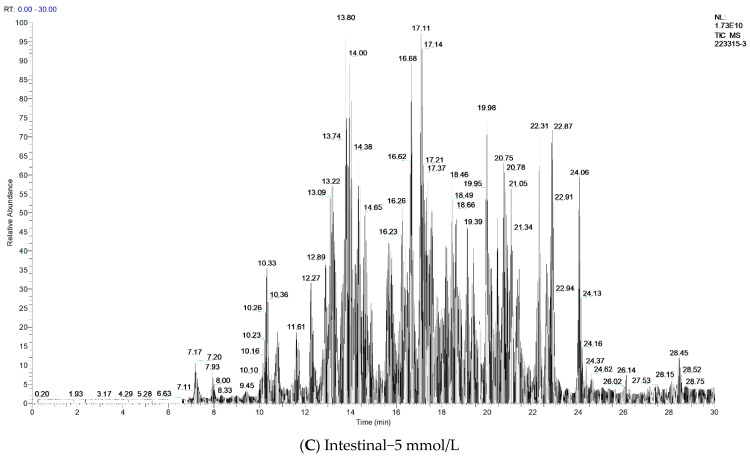
Total ions (X: time, Y: intensity) of walnut protein samples. (**A**) Sample of gastric digestion that is not oxidized; (**B**) sample of gastric digestion at the AAPH concentration of 5 mmol/L; and (**C**) sample of subsequent intestinal digestion at the AAPH concentration of 5 mmol/L.

We later characterized the representative samples of G-0, G-5, and I-5 by using the LC-MS/MS technology. The identification results are shown in Table 2. In three samples, it obtained 91, 534, and 450 peptide sequences and identified 22, 31, and 25 proteins, respectively. The major proteins identified as resistant to gastric and intestinal digestion from the detected peptides were the 11S globulin seed storage protein, legumin, vicilin, 2S sulfur-rich seed storage protein, and other important seed storage proteins, such as oleosin, actin, glutaredoxin, salicylate carboxymethyltransferase-like, and ubiquitin-40S ribosomal protein S27a-like. It found that aquaporin, cell wall structural proteins, histone, ankyrin repeat-containing proteins, and some enzymes were resistant to pepsin after oxidative modification, but no corresponding peptides were detected at G-0 and GI-5. It is possible that the oxidative modifications are resistant to digestion in the stomach and may be digested further in the intestine, or that the oxidatively modified proteins promote trypsin hydrolysis, which corresponds to the results shown by Tricine-SDS-PAGE in the intestinal phase. As shown in the total ion chromatography in Figure 6, it can also be clearly seen that oxidation affects the digestibility of proteins, among which the sample of G-0 has the lowest detected abundance, indicating that the unoxidized sample has a higher degree of digestion, and G-5 and I-5 were less digested than the unoxidized sample based on the higher detected abundance. Subsequently, we performed a peptide sequence summary of the top 10 proteins identified, the results of which are available in the Supplementary File (Document S1). The gray-labeled sequences are the same peptide sequences as G-0, G-5, and I-5. From the sequence comparison of G-0 and G-5, it can be found that a large number of new peptides are produced by oxidation during gastric digestion, and some peptides are increased in the intestine, which indicates that oxidation may cause cross-linked aggregation, mask the pepsin recognition site, and thus produce resistance to gastric digestion.

## 4. Conclusions

Alkoxy radical oxidation induced by AAPH causes a series of structural changes in proteins, such as a decrease in free amino groups, a loss of sulfhydryl groups, decreased surface hydrophobicity, and increased tryptophan metabolite NFk content and Schiff base content. In amino acid analysis, before and after oxidation and digestion, cysteine is usually the first amino acid to be oxidized, which also implies the importance of the disulfide bond in protein aggregates. Protein oxidation unfolding and aggregation were found to occur simultaneously in SDS-PAGE profiles, and the Cys-S-S-linking generated from the cysteine and free sulfhydryl group reactions and the increased content of NFk and Schiff bases may all be responsible for the soluble aggregation.

Protein oxidation leads to an increase in the carbonyl content, and the generation of the carbonyl groups is a complex process often associated with protein cross-linking and forming aggregates, which has an impact on digestion. The major proteins identified as resistant to gastric and intestinal digestion from the detected peptides were mostly seed storage protein. As a result, it is critical to investigate the properties of the oxidation and digestion products of walnut protein structure. The mechanism of walnut protein oxidation and digestion is beneficial to the quality of the protein processing process because it provides the foundation and direction for research into the digestion characteristics of walnut-based complex proteins.

## Figures and Tables

**Table 1:** Effects of oxidation on the carbonyl group, free sulfhydryl group, and free amino group of walnut samples with increasing concentrations of AAPH ^a^.

AAPH (mmol/L)	Carbonyl (nmol/mg)	Free SH (nmol/mg)	Free Amino (μmol/mg)
0	2.36 ± 0.09 f	4.30 ± 0.03 a	1.28 ± 0.03 c
0.04	2.42 ± 0.11 e	3.80 ± 0.05 b	1.39 ± 0.01 b
0.2	2.68 ± 0.05 d	3.66 ± 0.02 c	1.47 ± 0.02 a
1	3.18 ± 0.02 c	2.22 ± 0.01 d	1.17 ± 0.01 d
5	4.23 ± 0.07 b	2.02 ± 0.01 e	1.15 ± 0.02 d
25	5.12 ± 0.10 a	1.72 ± 0.08 f	1.13 ± 0.02 d

^a^ Each index of the sample is the average of three determinations, and the data are expressed as mean ± standard error. Different letters in the same column indicate significant differences at the *p* < 0.05 level.

**Table 2:** The identification results of walnut protein digestion products (G-0, G-5, and I-5) by LC-MS/MS G-0: gastric digestion that is not oxidized; G-5: gastric digestion at the AAPH concentration of 5 mmol/L; I-5: subsequent intestinal digestion at the AAPH concentration of 5 mmol/L; UP: unique peptides.

	ACCESSION	DESCRIPTION	MW	PI	G-0		G-5		I-5	
					UP	SCORE	UP	SCORE	UP	SCORE
1	XP_018818401.1	11S globulin seed storage protein 2-like	54.3	6.81	11	40.43	62	9.91	63	965.24
2	XP_018827137.1	11S globulin seed storage protein 2-like	53.4	7.8	2	8.39	20	5.51	18	186.72
3	XP_018827329.1	11S globulin seed storage protein Jug r 4	58.1	7.25	5	14.63	46	38.36	52	757.22
4	XP_018827328.1	11S globulin-like	58.3	7.77	13	45.10	66	119.56	59	908.08
5	XP_018842296.1	legumin B-like	55.8	7.5	12	74.54	71	12.29	64	1504.61
6	XP_018812171.1	vicilin Car i 2.0101	94.3	6.83	9	24.86	23	1048.8	13	73.32
7	XP_018814692.1	Vicilin Jug r 6.0101	57.4	7.3	5	19.99	22	30.3	15	73.04
8	XP_035546314.1	vicilin-like seed storage protein At2g28490	59.6	6.92	2	5.38	6	199.66	5	17.19
9	XP_018824007.1	2S sulfur-rich seed storage protein 2	17.1	6.52	5	27.03	3	2.7	3	65.45
10	XP_018837699.1	oleosin 18.2 kDa-like	16.6	9.91	2	7.39	4	2.63	3	5.78
11	XP_018842295.1	legumin B-like	55.8	8.18	1	28.82	2	21	2	399.8
12	XP_018856265.1	oleosin 1-like	14.7	10.14	4	8.72	1	2.2	1	2.92
13	XP_018842385.1	oleosin 5-like	16.3	10.08	2	5.3	5	0	2	2.46
14	XP_018814488.1	actin-97	41.7	5.49	3	6.52	5	7.3	3	8.54
15	XP_018821252.1	glutaredoxin-like	13.7	6.09	1	2.05	2	2.15	1	1.73
16	XP_035549483.1	salicylate carboxymethyltransferase-like	40.7	6.54	1	2.39	1	4.65	1	2.35
17	XP_018805239.1	ubiquitin-40S ribosomal protein S27a-like	17.7	9.79	1	2.09	2	3.09	1	2.87
18	XP_018810059.1	peroxygenase-like	26.9	5.87			2	2.6	2	2.17
19	XP_018831031.1	oleosin 1	14.8	9.63			1	0	1	2.62
20	XP_018809740.1	oleosin 18.2 kDa-like	15.9	9.72			3	0	1	0
21	XP_018818883.1	haloacid dehalogenase-like hydrolase domain-containing protein Sgpp	33.7	5.63	1	2.23			1	1.96
22	XP_018841715.1	histone H4-like	14.2	10.84	2	6.31	1	2.17		
23	XP_018812582.1	40S ribosomal protein S8-like	25.4	10.43	1	1.66				
24	XP_018845470.1	oleosin	15.6	9.54	1	1.94				
25	XP_018811862.1	aspartic proteinase-like isoform X1	57.9	5.39	1	2.04				
26	XP_018851532.1	probable aquaporin TIP3-2	27.4	7.71			1	5.54		
27	XP_018849277.1	probable aquaporin TIP3-2	27.4	7.66			1	3.21		
28	XP_018822092.1	protein FAM135B-like	91.8	8			1	874.88		
29	XP_035550528.1	putative glycine-rich cell wall structural protein 1 isoform X1	21.3	9.11			1	2.56		
30	XP_018841706.1	histone H2A	15.9	10.67			1	1.74		
31	XP_035543376.1	glyceraldehyde-3-phosphate dehydrogenase GAPCP2, chloroplastic-like	45.4	9.01			1	5.1		
32	XP_018826340.2	vicilin-like seed storage protein At2g18540	79	5.44			1	498.72		
33	XP_018811454.1	11-beta-hydroxysteroid ehydrogenase B-like	40.9	6.35			2	2.1		
34	XP_018809799.1	ankyrin repeat-containing protein ITN1-like	25.6	7.46			1	2.79		
35	XP_018821986.2	malate synthase, glyoxysomal	64.2	7.55			1	128.13		
36	XP_018835846.1	polygalacturonase QRT3	52.5	6.54					1	0
37	XP_018847613.1	histone H2AX-like	14.9	10.36					2	4.05
38	XP_018826287.1	barwin-like	20.4	7.62					1	0
39	XP_018828748.1	elongation factor 1-alpha	49.5	9.07					1	2.35

## Data Availability

Data is contained within the article or Supplementary Material.

## Supplementary Materials

The following supporting information can be downloaded at: https://www.mdpi.com/article/10.3390/foods11244104/s1, Document S1: A peptide sequence summary of the top 10 proteins identified.
