# Supplementary material for: Effect of Oxidative Modification by Peroxyl Radical on the Characterization and Identification of Oxidative Aggregates and In Vitro Digestion Products of Walnut (Juglans regia L.) Protein Isolates
Source: Foods. 2022 Dec 19;11(24):4104. doi: 10.3390/foods11244104 (PMC9777859; doi:10.3390/foods11244104)
Supplement: Supplementary file 1 [file foods-11-04104-s001.zip › foods-2059318-supplementary.pdf]

Document S1: A peptide sequence summary of the top 10 proteins identified.

G-O（Gastric-Native）

XP_018812171.1 vicilin Car i 2.0101 [312-321] [417-433] [469-485] [511-533] [635-647] [710-722] [749-768]

DRQDPQQQYHRCQRRCQIQEQSPERQRQCQQRCE
RQYKEQQGRERGPEASPRRESRGREEEQQRHNPY
YFHSQSIRSRHESEEGEVKYLERFTERTELLRGIEN
YRVVILDANPNTFMLPHHKDAESVAVVTRGRATLT
LVSQETRESFNLECGDVIRVPAGATVYYVINQDSNER
LEMVKLLQPVNNPGQFREYYAAGAKSPDQSYLRV
FSNDILVAALNTPRDRLERFFDQQEQREGVIIRASQ
EKLRALSQHAMSAGQRPWGRRSSGGPISLKSERPS
YSNQFGQFFEACPEEHRQLQEMDVLVNYAEIKRG
AMMVPHYNSKATVVVYVVEGTGRYEMACPHVSS
QSYEGQGRREQEEEESTGRFQKVTARLARGDIFVI
PAGHPIAITASQNENLRLLGFGINGENNQRNFLAG
QNNIINQLEREAKELSFNMPREEIEEIFESQMESYF
VPTERQS

XP_035546314.1 vicilin-like seed storage protein At2g28490 [50-70] [193-203]

EERKEREQRRSEREEGEEETDDRFLLQDSKSVMRT
EAGEMRVIKSLGGKIWDRPLHIGFITMEPQTLFIPQ
YLDSSLMIFIRRGEAKIGLIYKDELGERRLKTGDLY
RIPAGSAFYLVNTAEGQRLHIICSIDPSESLGIGTFQS
FFIGGGKYPTSVLAGFERETLSNAF

XP_018827328.1 11S globulin-like [43-51] [115-127] [178-217] [286-299] [332-364] [435-459] [465-479]

KRLVALEPSNRIEAEAGVIESWDPNNQQFQCAGVA
VVRRTIEPNGLLLPQYSNAPQLLYIVKGRGITGVLF
PGCPETFEESQQGQSRIRPSLRSASFQRDRHQKIRH
FREGDVIAFPAGVAHWCYNDGDTPVVTVALMDTT
NNANQLDQNPRNFYLAGNPDDEFRQEGQQEYEQ
HRRQQQHQQRHGEPGQQQRGSSNNVFSGFDAEFL
ADAFNVDTETARRLQSNHDQRRGIVRVEGNLQVI
RPRWSREEQELEDRREERQRELEWERERRQSRRTG
GRFDNGLEETVCTLRLRENIGDPSRADIYTEQAGRI
STVNSQNLPILRWLQLSAERGALYSDALYVPHWN
LNAHSVVYAIRGRAEIQVVDHLGQTVFDDDLREG
QLLTIPQNFGVVKRATGEGFEWVSFKTNENAMISP
LAGRTSAIRAIPEEVLANAFQIPRE

XP_018827329.1 11S globulin seed storage protein Jug r 4 [273-280] [346-358] [431-453]

GRQLQVIRPRWSREEQEREERKERERERESESERR
QSRRGGRDDNGLEETICTLRLRENIGDPSRADIYTE
EAGRISTVNSHTLPVLRWLQLSAERGALYSDALYV
PHWNLNAHSVVYALRGRAEVQVVDNFGQTVFDD
ELREGQLLTIPQNFAVVKRARNEGFEWVSFKTNEN
AMVSPLAGRTSAIRAL

G-5（Gastric-Oxidation）

XP_018812171.1 vicilin Car i 2.0101 [311-321] [327-335] [375-401] [417-443] [446-456] [469-502] [519-533] [539-546] [608-634] [655-665] [687-737]

DRQDPQQQYHRCQRRCQIQEQSPERQRQCQQRCE
RQYKEQQGRERGPEASPRRESRGREEEQQRHNPY
YFHSQSIRSRHESEEGEVKYLERFTERTELLRGIEN
YRVVILDANPNTFMLPHHKDAESVAVVTRGRATLT
LVSQETRESFNLECGDVIRVPAGATVYYVINQDSNER
LEMVKLLQPVNNPGQFREYYAAGAKSPDQSYLRV
FSNDILVAALNTPRDRLERFFDQQEQREGVIIRASQ
EKLRALSQHAMSAGQRPWGRRSSGGPISLKSERPS
YSNQFGQFFEACPEEHRQLQEMDVLVNYAEIKRG
AMMVPHYNSKATVVVYVVEGTGRYEMACPHVSS
QSYEGQGRREQEEEESTGRFQKVTARLARGDIFVI
PAGHPIAITASQNENLRLLGFGINGENNQRNFLAG
QNNIINQLEREAK

XP_035546314.1 vicilin-like seed storage protein At2g28490 [128-139] [149-165] [226-239] [392-402] [413-441]

IRRGEAKIGLIYKDELGERRLKTGDLYRIPAGSAFY
LVNTAEGQRLHIICSIDPSESLGIGTFQSFFIGGGKY
PTSVLAGFERETLSNAFNVSFSEVQDILSRQREGPI
VYVPESHSPSVWSKFLQMKERDRLQHLKKLVDFH
EPRDDDEEEEEEQQQQITWSWRKLLNSVLGKEPS
KRGDKRTRRTPDSYNLYKRRPDFRNNYGWSVALD
ESDYTPLKHSGISVFLVNLTAGSMMAPHVNPTATE
YGIVLSGSGTIQIVYPNGTSAMNAKISEGDVFWVP
KYFPFCQIASRTGPLEFFGFTTSARTNRPQFLVGAS
SILRTMLGPELAA

XP_018827328.1 11S globulin-like [42-51] [79-105] [115-127] [170-217] [247-260] [270-299] [332-459] [464-481] [487-496]

KRLVALEPSNRIEAEAGVIESWDPNNQQFQCAGVA
VVRRTIEPNGLLLPQYSNAPQLLYIVKGRGITGVLF
PGCPETFEESQQGQSRIRPSLRSASFQRDRHQKIRH
FREGDVIAFPAGVAHWCYNDGDTPVVTVALMDTT
NNANQLDQNPRNFYLAGNPDDEFRQEGQQEYEQ
HRRQQQHQQRHGEPGQQQRGSSNNVFSGFDAEFL
ADAFNVDTETARRLQSNHDQRRGIVRVEGNLQVI
RPRWSREEQELEDRREERQRELEWERERRQSRRTG
GRFDNGLEETVCTLRLRENIGDPSRADIYTEQAGRI
STVNSQNLPILRWLQLSAERGALYSDALYVPHWN
LNAHSVVYAIRGRAEIQVVDHLGQTVFDDDLREG
QLLTIPQNFGVVKRATGEGFEWVSFKTNENAMISP
LAGRTSAIRAIPEEVLANAFQIPRED
ARRLKFSRQETFLARSRSRSSTDIRRVVE

XP_018827329.1 11S globulin seed storage protein Jug r 4 [41-49] [77-100] [113-135] [174-211] [239-254] [273-280] [326-453] [458-473] [479-490]

MAKPILLSIYLFLIVALFNGCLAQSGGRQQQQFGQ
CQLNRLDALEPTNRIEAEAGVIESWDPNNQQFQC
AGVAVVRRTIEPNGLLLPQYSNAPQLVYIARGRGIT
GVLFPGCPETFEESQRQSQQGQSREFQQDRHQKIR
HFREGDIIAFPAGVAHWSYNDGSNPVVAISLLDTN
NNANQLDQNPRNFYLAGNPDDEFRPQGQQEYEQ
HRRQQQRQQRPGEHGQQQRGLGNNVFSGFDADF
LADAFNVDTETARRLQSENDHRRSIVRVEGRQLQ
VIRPRWSREEQEREERKERERERESESERRQSRRG
GRDDNGLEETICTLRLRENIGDPSRADIYTEEAGRI
STVNSHTLPVLRWLQLSAERGALYSDALYVPHWN
LNAHSVVYALRGRAEVQVVDNFGQTVFDDELRE
GQLLTIPQNFAVVKRARNEGFEWVSFKTNENAMV
SPLAGRTSAIRALPEEVLATAFQIPREDARRLKFNR
QESTLVRSRPSRSRSSRSERRAEV

I-5（Intestinal-Oxidation）

XP_018812171.1 vicilin Car i 2.0101[124-131] [375-386] [469-502] [511-533] [539-546] [624-634] [655-665] [687-737]

CERQFQEQEERQRQCQQRCERQYKEQQGRERGPE
ASPRRESRGREEEQQRHNPYYFHSQSIRSRHESEE
GEVKYLERFTERTELLRGIENYRVVILDANPNTFM
LPHHKDAESVAVVTRGRATLTLVSQETRESFNLEC
GDVIRVPAGATVYYVINQDSNERLEMVKLLQPVNN
PGQFREYYAAGAKSPDQSYLRVFSNDILVAALNTP
RDRLERFFDQQEQREGVIIRASQEKLRALSQHAMS
AGQRPWGRRSSGGPISLKSERPSYSNQFGQFFEAC
PEEHRQLQEMDVLVNYAEIKRGAMMVPHYNSKA
TVVVYVVEGTGRYEMACPHVSSQSYEGQGRREQ
EEEESTGRFQKVTARLARGDIFVIPAGHPIAITASQN
ENLRLLGFGINGENNQRNFLAGQNNIINQLEREAK

XP_035546314.1 vicilin-like seed storage protein At2g28490 [50-70] [128-139] [149-165] [413-441]

RSEREEGEEETDDRFLLQDSKSVMRTEAGEMRVIK
SLGGKIWDRPLHIGFITMEPQTLFIPQYLDSSLMIFI
RRGEAKIGLIYKDELGERRLKTGDLYRIPAGSAFYL
VNTAEGQRLHIICSIDPSESLGIGTFQSFFIGGGKYP
TSVLAGFERETLSNAFNVSFSEVQDILSRQREGPIV
YVPESHSPSVWSKFLQMKERDRLQHLKKLVDFHE
PRDDDEEEEEEQQQQITWSWRKLLNSVLGKEPSK
RGDKRTRRTPDSYNLYKRRPDFRNNYGWSVALDE
SDYTPLKHSGISVFLVNLTAGSMMAPHVNPTATEY
GIVLSGSGTIQIVYPNGTSAMNAKISEGDVFWVPK
YFPFCQIASRTGPLEFFGFTTSARTNRPQFLVGASSI
LRTMLGPELAA

XP_018827328.1 11S globulin-like [42-51] [80-105] [115-127] [179-216] [234-261] [270-299] [332-378] [398-459] [464-484] [487-496]

KRLVALEPSNRIEAEAGVIESWDPNNQQFQCAGVA
VVRRTIEPNGLLLPQYSNAPQLLYIVKGRGITGVLF
PGCPETFEESQQGQSRIRPSLRSASFQRDRHQKIRH
FREGDVIAFPAGVAHWCYNDGDTPVVTVALMDTT
NNANQLDQNPRNFYLAGNPDDEFRQEGQQEYEQ
HRRQQQHQQRHGEPGQQQRGSSNNVFSGFDAEFL
ADAFNVDTETARRLQSNHDQRRGIVRVEGNLQVI
RPRWSREEQELEDRREERQRELEWERERRQSRRTG
GRFDNGLEETVCTLRLRENIGDPSRADIYTEQAGRI
STVNSQNLPILRWLQLSAERGALYSDALYVPHWN
LNAHSVVYAIRGRAEIQVVDHLGQTVFDDDLREG
QLLTIPQNFGVVKRATGEGFEWVSFKTNENAMISP
LAGRTSAIRAIPEEVLANAFQIPREDARRLKFSRQE
TFLARSRSR

XP_018827329.1 11S globulin seed storage protein Jug r 4 [40-49] [77-100] [113-135] [174-211] [239-254] [281-291] [326-427] [429-473] [479-490]

QQFGQCQLNRLDALEPTNRIEAEAGVIESWDPNN
QQFQCAGVAVVRRTIEPNGLLLPQYSNAPQLVYIA
RGRGITGVLFPGCPETFEESQRQSQQGQSREFQQD
RHQKIRHFREGDIIAFPAGVAHWSYNDGSNPVVAIS
LLDTNNNANQLDQNPRNFYLAGNPDDEFRPQGQ
QEYEQHRRQQQRQQRPGEHGQQQRGLGNNVFSG
FDADFLADAFNVDTETARRLQSENDHRRSIVRVEG
RQLQVIRPRWSREEQEREERKERERERESESERRQ
SRRGGRDDNGLEETICTLRLRENIGDPSRADIYTEE
AGRISTVNSHTLPVLRWLQLSAERGALYSDALYVP
HWNLNAHSVVYALRGRAEVQVVDNFGQTVFDDE
LREGQLLTIPQNFAVVKRARNEGFEWVSFKTNENA
MVSPLAGRTSAIRALPEEVLATAFQIPREDARRLKF
NRQESTLVRSRPSRSRSSR

XP_018814692.1 Vicilin Jug r 6.0101 [93-111] [308-317] [407-427] [452-463]

Document S1: A peptide sequence summary of the top 10 proteins identified.

LGGEREEENPYVFEDEDFETRVRTDEGRIQVLEKFTKRSKLLRGIENFRVAILEANPQTFISPAHFDAELVVFVAKGRATITTVREEKRENFNVEQGDIMRIPAGTPVYLINRDENEKLYIVKILRPVSVPGHFEAFHGSGGEDPESFYRAFSWEVLEAALKTRRDQLEKLFGKQTQGVIIKASKEQIRSMSKHEETTPRIWPFGGDSTHPFNLFHKRPSQSNQFGRLFETDPKECKQLQDLDLMVSFANITKGSMAGPYYNSRATKISVVIEGEGYFEMACPHLSSSGSRGQREGSGSSRRRSRSGPSYQQIRGRLRPGMVFVAPAGHPVAVIASRNKNLQVLCFDVNAQGNIRFPLAGKNNIVNEFEKEAKELAFNFP

XP_018842296.1 legumin B-like [45-69] [89-101] [179-195] [197-211] [274-284] [361-380] [385-429] [448-459] [473-487]

TECRINNLNAQEPGRRVESEAGLSEYWNRNDEQFRCAGVDLVRHTIQRRGLLLPSFSNAPRLVYVVQGRGLHGAAIPGCPETFQSESSSQFRGEQGSQRLSRDQHQKVREIREGDVVAIPAGVAHWIYNDGESQLIVMILYDTSNQANQLDENARRFYLAGNPHQQQQGGRQRRRESWPGSRSRSRSQSPEERERSQQSGSNIFSGFDEEFLADSFNIDNELAMRIQNRDDQRGIIVTVEDELRVLSPQSRGEEREERGLDRDNGVEETFCTLRLKHNIADAQRADVYNERGGRITSLNSFNLPILRYIQLSAERGVLYRNALVAPHYNLNSHSVIYVIRGNARLQVVGENGQNVFDGEIREGQALTVPQNFAVIKKAGNQGFEWVAFKTNDNAKINALAGRLSTMRALPEDVLINAYRIDREEARRLKYNRDETTLFSSDSSGSPRRRD

XP_018818401.1 11S globulin seed storage protein 2-like [111-124] [182-195] [284-297] [313-326] [398-425] [433-459] [469-479]

AETYESETSQDLSRRRSSQMGQLQGGGDQQRSRRADQHQKVNRIRRGDIVAIPAGVAHWSYNDGNEELVAFAVIDLSNHANQLDRRFRSFLLAGGEPRDGQSGQGGSRQEERQEQRSQRSRREQRGSFQNIFSGFSEELLAEAYNIPDTIARRLQEDDNRRGVIVKCQDEMRRMMRPDEDEQEGQRQLVNGLEETLCTTRIRHNLDTQTESDVFSRQAGRVNIVNQHKLPILRYLDMSAEKGHLFPNALYTPHWSMTDNRVVYVLRGDARVQIVDDNGDNVFDERVKRGDVYVIPQFYATTARAGNNGFEYVTIKTSGQPMKSPMAGYTSVIRAMPIDVLTNSFQMSPREAQNLKHNRGHQSFLLSSSRSS

XP_018827137.1 11S globulin seed storage protein 2-like [369-383] [457-467]

YVLRGEARVQIVDDNGNNVFDERVKRGDVFVIPQFFAVTSKAGNDGFEYVTIKTSGQPMKSPMAGYTSVIKAMPIDVLANAYQMSLRDAQNLKNSRGHQSFLLSSSRSAS

XP_018824007.1 2S sulfur-rich seed storage protein 2 [106-126]

RQVVRRQQQQQGLRGEEMEEMVQSARDLPN

XP_018837699.1 oleosin 18.2 k Da-like [113-123] [125-140]

RRTRVPEQLEHAKRRAHEAAGQMGQTVTGK

XP_018814692.1 Vicilin Jug r 6.0101 [96-111] [169-254] [309-317] [396-404] [407-427] [464-472] [480-492]

LGGEREEENPYVFEDEDFETRVRTDEGRIQVLEKFTKRSKLLRGIENFRVAILEANPQTFISPAHFDAELVVFVAKGRATITTVREEKRENFNVEQGDIMRIPAGTPVYLINRDENEKLYIVKILRPVSVPGHFEAFHGSGGEDPESFYRAFSWEVLEAALKTRRDQLEKLFGKQTQGVIIKASKEQIRSMSKHEETTPRIWPFGGDSTHPFNLFHKRPSQSNQFGRLFETDPKECKQLQDLDLMVSFANITKGSMAGPYYNSRATKISVVIEGEGYFEMACPHLSSSGSRGQREGSGSSRRRSRSGPSYQQIRGRLRPGMVFVAPAGHPVAVIASRNKNLQVLCFDVNAQGNIRFPLAGKNNIVNEFEKEAKELAFNFPAREVEKIFRNQDQEFFFPGPSRQPEEGGRA

XP_018842296.1 legumin B-like [23-33] [44-75] [89-139] [179-211] [274-284] [305-381] [385-459] [467-487]

LAQLEQVTGQSQRQQQQRFQTECRINNLNAQEPGRRVESEAGLSEYWNRNDEQFRCAGVDLVRHTIQRRGLLLPSFSNAPRLVYVVQGRGLHGAAIPGCPETFQSESSSQFRGEQGSQRLSRDQHQKVREIREGDVVAIPAGVAHWIYNDGESQLIVMILYDTSNQANQLDENARRFYLAGNPHQQQQGGRQRRRESWPGSRSRSRSQSPEERERSQQSGSNIFSGFDEEFLADSFNIDNELAMRIQNRDDQRGIIVTVEDELRVLSPQSRGEEREERGLDRDNGVEETFCTLRLKHNIADAQRADVYNERGGRITSLNSFNLPILRYIQLSAERGVLYRNALVAPHYNLNSHSVIYVIRGNARLQVVGENGQNVFDGEIREGQALTVPQNFAVIKKAGNQGFEWVAFKTNDNAKINALAGRLSTMRALPEDVLINAYRIDREEARRLKYNRDETTLFSSDSSGSPRRRD

XP_018818401.1 11S globulin seed storage protein 2-like [111-141] [156-173] [182-196] [199-208] [311-481]

GLLGLTYPGCAETYESETSQDLSRRRSSQMGQLQGGGDQQRSRRADQHQKVNRIRRGDIVAIPAGVAHWSYNDGNEELVAFAVIDLSNHANQLDRRFRSFLLAGGEPRDGQSGQGGSRQEERQEQRSQRSRREQRGSFQNIFSGFSEELLAEAYNIPDTIARRLQEDDNRRGVIVKCQDEMRRMMRPDEDEQEGQRQLVNGLEETLCTTRIRHNLDTQTESDVFSRQAGRVNIVNQHKLPILRYLDMSAEKGHLFPNALYTPHWSMTDNRVVYVLRGDARVQIVDDNGDNVFDERVKRGDVYVIPQFYATTARAGNNGFEYVTIKTSGQPMKSPMAGYTSVIRAMPIDVLTNSFQMSPREAQNLKHNRGHQSFLLSSSRSS

XP_018827137.1 11S globulin seed storage protein 2-like [195-204] [299-314] [319-326] [332-339] [348-467]

DRRFRSFFLAGGEPRQEHMEGDRERTRQRRSRQQRVGFQSLFSGFSEELLAEAYNIPVNIARRLQEDDSQRGIIVRCQEEMRRMIRPDEDRQEGQRWLNGLEETVCTTRIRHNLDTQSESDFVSRQGGRVNIVNMHKLPILRFMDMSAEKGHLFPNAMYTPHWSMTDNRVVYVLRGEARVQIVDDNGNNVFDERVKRGDVFVIPQFFAVTSKAGNDGFEYVTIKTSGQPMKSPMAGYTSVIKAMPIDVLANAYQMSLRDAQNLKNSRGHQSFLLSSSRSAS

XP_018824007.1 2S sulfur-rich seed storage protein 2 [106-126]

RQVVRRQQQQQGLRGEEMEEMVQSARDLPN

XP_018837699.1 oleosin 18.2 k Da-like [101-112]

RRTRVPEQLEHAKRRAHEAA

XP_018814692.1 Vicilin Jug r 6.0101 [169-252] [265-272] [308-317] [344-354] [407-427] [452-460] [464-472] [480-492]

VVFVAKGRATITTVREEKRENFNVEQGDIMRIPAGTPVYLINRDENEKLYIVKILRPVSVPGHFEAFHGSGGEDPESFYRAFSWEVLEAALKTRRDQLEKLFGKQTQGVIIKASKEQIRSMSKHEETTPRIWPFGGDSTHPFNLFHKRPSQSNQFGRLFETDPKECKQLQDLDLMVSFANITKGSMAGPYYNSRATKISVVIEGEGYFEMACPHLSSSGSRGQREGSGSSRRRSRSGPSYQQIRGRLRPGMVFVAPAGHPVAVIASRNKNLQVLCFDVNAQGNIRFPLAGKNNIVNEFEKEAKELAFNFPAREVEKIFRNQDQEFFFPGPSRQPEEGGRA

XP_018842296.1 legumin B-like
[23-33] [44-75] [89-133] [179-211] [274-291] [303-331] [335-347] [356-417] [430-487]

CLAQLEQVTGQSQRQQQQRFQTECRINNLNAQEPGRRVESEAGLSEYWNRNDEQFRCAGVDLVRHTIQRRGLLLPSFSNAPRLVYVVQGRGLHGAAIPGCPETFQSESSSQFRGEQGSQRLSRDQHQKVREIREGDVVAIPAGVAHWIYNDGESQLIVMILYDTSNQANQLDENARRFYLAGNPHQQQQGGRQRRRESWPGSRSRSRSQSPEERERSQQSGSNIFSGFDEEFLADSFNIDNELAMRIQNRDDQRGIIVTVEDELRVLSPQSRGEEREERGLDRDNGVEETFCTLRLKHNIADAQRADVYNERGGRITSLNSFNLPILRYIQLSAERGVLYRNALVAPHYNLNSHSVIYVIRGNARLQVVGENGQNVFDGEIREGQALTVPQNFAVIKKAGNQGFEWVAFKTNDNAKINALAGRLSTMRALPEDVLINAYRIDREEARRLKYNRDETTLFSSDSSGSPRRRD

XP_018818401.1 11S globulin seed storage protein 2-like [111-124] [127-141] [156-196] [199-218] [284-297] [311-376] [381-481]

AETYESETSQDLSRRRSSQMGQLQGGGDQQRSRRADQHQKVNRIRRGDIVAIPAGVAHWSYNDGNEELVAFAVIDLSNHANQLDRRFRSFLLAGGEPRDGQSGQGGSRQEERQEQRSQRSRREQRGSFQNIFSGFSEELLAEAYNIPDTIARRLQEDDNRRGVIVKCQDEMRRMMRPDEDEQEGQRQLVNGLEETLCTTRIRHNLDTQTESDVFSRQAGRVNIVNQHKLPILRYLDMSAEKGHLFPNALYTPHWSMTDNRVVYVLRGDARVQIVDDNGDNVFDERVKRGDVYVIPQFYATTARAGNNGFEYVTIKTSGQPMKSPMAGYTSVIRAMPIDVLTNSFQMSPREAQNLKHNRGHQSFLLSSSRSS

XP_018827137.1 11S globulin seed storage protein 2-like [195-204] [299-314] [319-326] [340-358] [369-447]

RRFRSFFLAGGEPRQEHMEGDRERTRQRRSRQQRVGFQSLFSGFSEELLAEAYNIPVNIARRLQEDDSQRGIIVRCQEEMRRMIRPDEDRQEGQRWLNGLEETVCTTRIRHNLDTQSESDFVSRQGGRVNIVNMHKLPILRFMDMSAEKGHLFPNAMYTPHWSMTDNRVVYVLRGEARVQIVDDNGNNVFDERVKRGDVFVIPQFFAVTSKAGNDGFEYVTIKTSGQPMKSPMAGYTSVIKAMPIDVLANAYQMSLRDAQ

XP_018824007.1 2S sulfur-rich seed storage protein 2 [106-126]

RQVVRRQQQQQGLRGEEMEEMVQSARDLPN

XP_018837699.1 oleosin 18.2 k Da-like [113-123] [126-140]

RRTRVPEQLEHAKRRAHEAAGQMGQTVTGK
